# Oral anticoagulant re-initiation following intracerebral hemorrhage in non-valvular atrial fibrillation: Global survey of the practices of neurologists, neurosurgeons and thrombosis experts

**DOI:** 10.1371/journal.pone.0191137

**Published:** 2018-01-25

**Authors:** Yan Xu, Ashkan Shoamanesh, Sam Schulman, Dar Dowlatshahi, Rustam Al-Shahi Salman, Ioana Doina Moldovan, Philip Stephen Wells, Fahad AlKherayf

**Affiliations:** 1 Department of Medicine, University of Toronto, Toronto, Canada; 2 Division of Neurology, Department of Medicine, Population Health Research Institute, McMaster University, Hamilton, Canada; 3 Division of Hematology, Department of Medicine, McMaster University, Hamilton, Canada; 4 Division of Neurology, Department of Medicine, University of Ottawa, Ottawa, Canada; 5 Centre for Clinical Brain Sciences, University of Edinburgh, Edinburgh, United Kingdom; 6 Division of Neurosurgery, Department of Surgery, University of Ottawa, Ottawa, Canada; 7 Department of Medicine, University of Ottawa, Ottawa, Canada; Monash University, AUSTRALIA

## Abstract

**Background:**

While oral anticoagulants (OACs) are highly effective for ischemic stroke prevention in atrial fibrillation, intracerebral hemorrhage (ICH) remains the most feared complication of OAC. Clinical controversy remains regarding OAC resumption and its timing for ICH survivors with atrial fibrillation because the balance between risks and benefits has not been investigated in randomized trials.

**Aims/Hypothesis:**

To survey the practice of stroke neurologists, thrombosis experts and neurosurgeons on OAC re-initiation following OAC-associated ICH.

**Methods:**

An online survey was distributed to members of the International Society for Thrombosis and Haemostasis, Canadian Stroke Consortium, NAVIGATE-ESUS trial investigators (Clinicatrials.gov identifier NCT02313909) and American Association of Neurological Surgeons. Demographic factors and 11 clinical scenarios were included.

**Results:**

Two hundred twenty-eight participants from 38 countries completed the survey. Majority of participants were affiliated with academic centers, and >20% managed more than 15 OAC-associated ICH patients/year. Proportion of respondents suggesting OAC anticoagulant resumption varied from 30% (for cerebral amyloid angiopathy) to 98% (for traumatic ICH). Within this group, there was wide distribution in response for timing of resumption: 21.4% preferred to re-start OACs after 1–3 weeks of incident ICH, while 25.3% opted to start after 1–3 months. Neurosurgery respondents preferred earlier OAC resumption compared to stroke neurologists or thrombosis experts in 5 scenarios (p<0.05 by Kendall’s tau).

**Conclusions:**

Wide variations in current practice exist among management of OAC-associated ICH, with decisions influenced by patient- and provider-related factors. As these variations likely reflect the lack of high quality evidence, randomized trials are direly needed in this population.

## Introduction

Despite the efficacy of oral anticoagulation for ischemic stroke prevention among patients with non-valvular atrial fibrillation (AF) [1], intracranial hemorrhage remains the most feared complication of anticoagulant treatment. Anticoagulating AF patients after an intracerebral haemorrhage (ICH) poses a challenging clinical dilemma that requires balancing the benefit of reducing thromboembolism against the increased risk of recurrent ICH, given that both have high morbidity and mortality [2,3].

Previous ICH was an exclusion criterion for all randomized controlled trials (RCT) that tested anticoagulation in patients with AF. The lack of evidence to guide treatment decisions is reflected in the wide variation in practice patterns [4], with one international, multi-center study demonstrating a 4-fold difference in rates of oral anticoagulant (OAC) re-initiation at time of discharge following an ICH [5]. Another frequent challenge in management of anticoagulant-associated ICH is the timing of OAC re-initiation following stabilization. While recent guidelines from the American Stroke Association and European Society of Cardiology recommend restarting OAC among selected AF patients with ICH after a minimum of 4 weeks [6], the quality of evidence supporting this recommendation was poor, and it is uncertain whether this has translated into clinical practice.

Therefore, we sought to determine the current practice of stroke neurologists, thrombosis specialists and neurosurgeons regarding the timing of OAC re-initiation in adult patients with non-valvular AF who present with anticoagulant-associated ICH, as well as clinical factors that influence their decisions.

## Methods

### Study population

Between November 2015 and April 2016, we surveyed members of four organizations: International Society for Thrombosis and Haemostasis (ISTH) via its Scientific and Standardization Committee on Control of Anticoagulation, the Canadian Stroke Consortium, investigators participating in the NAVIGATE-ESUS trial, and the American Association of Neurological Surgeons (AANS). ISTH members include clinicians (hematologists, cardiologists and neurologists) with an interest in thrombotic diseases and specifically involved in clinical care and research related to anticoagulation. The Canadian Stroke Consortium consists of stroke neurologists involved in patient care and research. NAVIGATE-ESUS (ClinicalTrials.gov identifier: NCT02313909) is an international, multicenter, double-dummy, superiority phase III trial of rivaroxaban against aspirin in secondary prevention of stroke and systemic embolism in patients with recent embolic strokes of undetermined source. The trial investigators primarily included stroke neurologists. Active members of the AANS include neurosurgeons practicing general neurosurgery or with specialized interests in neurosurgical subspecialties.

### Survey

An online, cross-sectional survey was constructed via FluidSurvey. The first part of the questionnaire included 5 questions on respondents’ specialty, years of practice, country of practice, practice type and exposure to anticoagulant-associated intracerebral hemorrhage in patients with non-valvular AF. Respondents were then asked to answer several key questions following a hypothetical clinical vignette involving an anticoagulant-associated ICH, pertaining to 1) whether the respondent would recommend anticoagulant re-initiation; 2) if so, what agent/dose the respondent would select, and 3) how long the respondent would wait prior to restarting therapy. The scenario was further qualified by 11 clinical factors that may change a clinician’s decision on OAC resumption, including size and location of the hemorrhage, risk factors on neuroimaging, baseline ischemic stroke risk, and ICH associated with a direct oral anticoagulant (compared to warfarin). All answers were multiple choice with single response.

In addition, several clinician-level factors were collected to understand their influences on treatment decisions. Finally, respondents were asked whether they would be interested in enrolling patients into a randomized controlled trial or in a cohort study that aimed to address these clinical questions. The survey questions are listed in **S1 File**.

All data were initially collected and stored online via FluidSurvey, and only accessible to the survey administrator. Reminder e-mails were sent to eligible participants 1 month following the initial survey invitation.

### Data analysis

The outcome of interest was recommended timing of OAC resumption, and we considered participant characteristics and demographics (specialty, country of practice, years in practice, type of practice and ICH cases managed per year) as covariates. For descriptive analysis, univariate analysis was used to calculate variable characteristics at baseline, which were then stratified by covariates.

In addition, we performed bivariate analysis to examine the relationship of participant characteristics and demographics in response to the clinical scenarios. Kendall’s tau coefficient was used for ordinal variables in bivariate analysis to evaluate the impact of specialty type, years of practice and cases of ICH per year on timing of OAC initiation and frequency of neuro-imaging for risk stratification. Cramer’s V measure was used for nominal variables in the bivariate analysis to evaluate the impact of provider characteristics on OAC agent of choice. χ^2^ test was used to evaluate the impact of geographical distribution of respondents (North America compared to outside of North America) on responses. P-value of 0.05 was considered statistically significant. SPSS and Microsoft Excel were used for statistical analysis.

### Ethics

The survey was approved by the Ottawa Health Science Network Research Ethics Board. All potential participants were sent an introductory e-mail prior to a second e-mail with the survey link. Initiation of the survey implied consent to participate, and written consent was waived by the Research Ethics Board.

## Results

A total of 1704 potential participants were identified, consisting of 1076 stroke neurologists, 315 thrombosis experts and 313 neurosurgeons. 228 clinicians completed our survey for a response rate of 13.4%, balanced across the three specialties (**Table 1**). Fifty-one percent of respondents were practicing in the U.S. or Canada, with the remainder of respondents from a total of 36 countries. Most were affiliated with academic centers, and 20.2% managed >15 anticoagulant-associated ICH patients with non-valvular AF on an annual basis (**Table 1**).

**Table 1:** Demographic characteristics of survey respondents.

		% (n = 228)
Specialty	Stroke Neurologist	41.2
	Thrombosis	32.5
	Neurosurgeons	26.3
Country of Practice	United States	33.3
	Canada	18
	Other^a^	48.7
Years of Practice	0–5	18.9
	6–10	20.6
	11–15	12.3
	16–20	14.5
	21–25	16.2
	>25	17.5
Practice Setting	University	49.6
	University-Affiliated	24.1
	Community/Private	26.3
ICH Patients with non-valvular AF per year	0–5	33.3
	6–10	30.7
	11–15	15.8
	>15	20.2

^a^Largest nationalities represented included Italy (5.2%), Spain (5.2%), United Kingdom (3.9%), Argentina (3.5%), Russia (2.6%) and Brazil (2.2%).

ICH, intracranial hemorrhage.

### Timing of OAC re-initiation

**Fig 1:**
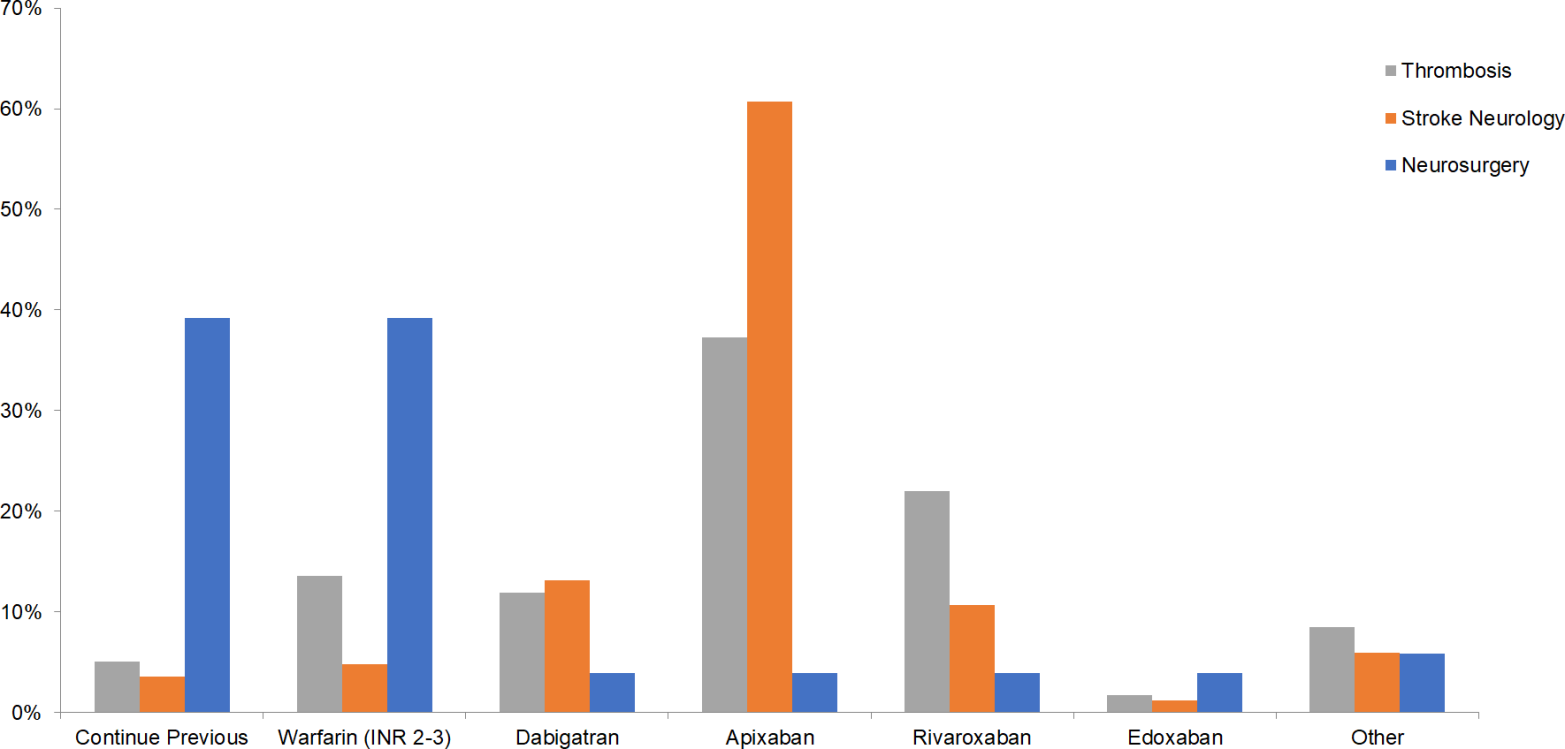
Overall response from survey participants on timing of OAC re-initiation across 11 clinical scenarios. ICH, intracerebral haemorrhage; DOAC, direct oral anticoagulants; IPH, intraparenchymal haemorrhage; HTN, hypertension; CHADS2, Congestive heart failure, hypertension, age (≥75), diabetes, stroke/TIA score.

Across all 11 clinical scenarios, two were associated with <50% rate of OAC re-initiation: presence of cerebral amyloid angiopathy on imaging (lobar ICH with ≥1 strictly lobar or cerebellar microbleeds, convexal subarachnoid hemorrhage and/or cortical superficial siderosis) [7] or prior ICH other than the index event; for whom the episode represented a second occurrence (**Fig 1**). Meanwhile, less than 20% of respondents opted for lifelong OAC cessation among cases with traumatic ICH, high ischemic stroke risk (CHADS_2_ = 5), small hematomas (<30 cm^2^) or deep intra-parenchymal hemorrhage with blood pressure control (**Fig 1**). Among respondents who elected to re-initiate OAC treatment, initiation of anticoagulation at 3–4 weeks (21.4%) and 1–3 months (25.3%) following the initial ICH were the two most frequently chosen timeframes.

Specialty-specific differences were observed among cases involving craniotomy, large hematoma >30 cm^2^, lobar hemorrhage, intra-parenchymal hemorrhage with blood pressure control and previous ICH (p<0.05 by Kendall’s tau). In all cases, neurosurgery respondents preferred to start OAC earlier than stroke neurologists or thrombosis experts (**Table 2**). No differences in decision or timing of OAC re-initiation was observed across participants’ years in practice, cases of OAC-associated ICH treated per year or geographic distribution.

**Table 2:** Specialty-specific variation in oral anticoagulant re-initiation following intracranial hemorrhage.

		Specialty		
		Stroke Neurology (n = 94)	Thrombosis (n = 74)	Neurosurgery (n = 60)
Craniotomy	Within 7 days	0.0%	4.20%	11.7%
	1–2 weeks	2.2%	15.50%	15.0%
	3–4 weeks	14.3%	12.70%	40.0%
	1–3 months	37.4%	25.40%	31.7%
	4–6 months	8.8%	7.00%	0.0%
	7–12 months	5.5%	2.80%	0.0%
	Never	31.9%	32.40%	1.7%
Large Hematoma (>30cm^2^)	Within 7 days	0.0%	4.30%	8.6%
	1–2 weeks	2.2%	8.60%	15.5%
	3–4 weeks	15.6%	18.60%	31.0%
	1–3 months	33.3%	22.90%	37.9%
	4–6 months	13.3%	7.10%	1.7%
	7–12 months	5.6%	2.90%	0.0%
	Never	30.0%	35.70%	5.2%
Lobar Hemorrhage	Within 7 days	0.0%	3.20%	9.3%
	1–2 weeks	1.1%	9.70%	18.5%
	3–4 weeks	20.5%	17.70%	33.3%
	1–3 months	20.5%	27.40%	31.5%
	4–6 months	8.0%	8.10%	0.0%
	7–12 months	2.3%	1.60%	0.0%
	Never	47.7%	32.30%	7.4%
Intraprenchymal haemorrhage with blood pressure control	Within 7 days	1.1%	4.80%	9.3%
	1–2 weeks	15.9%	11.10%	20.4%
	3–4 weeks	19.3%	23.80%	38.9%
	1–3 months	36.4%	25.40%	25.9%
	4–6 months	5.7%	6.30%	1.9%
	7–12 months	4.5%	3.20%	0.0%
	Never	17.0%	25.40%	3.7%
Previous ICH	Within 7 days	1.2%	3.40%	6.1%
	1–2 weeks	1.2%	5.10%	2.0%
	3–4 weeks	9.3%	10.20%	24.5%
	1–3 months	7.0%	5.10%	16.3%
	4–6 months	7.0%	3.40%	4.1%
	7–12 months	0.0%	0.00%	0.0%
	Never	74.4%	72.90%	46.9%

### OAC agent for re-initiation

**Fig 2:**
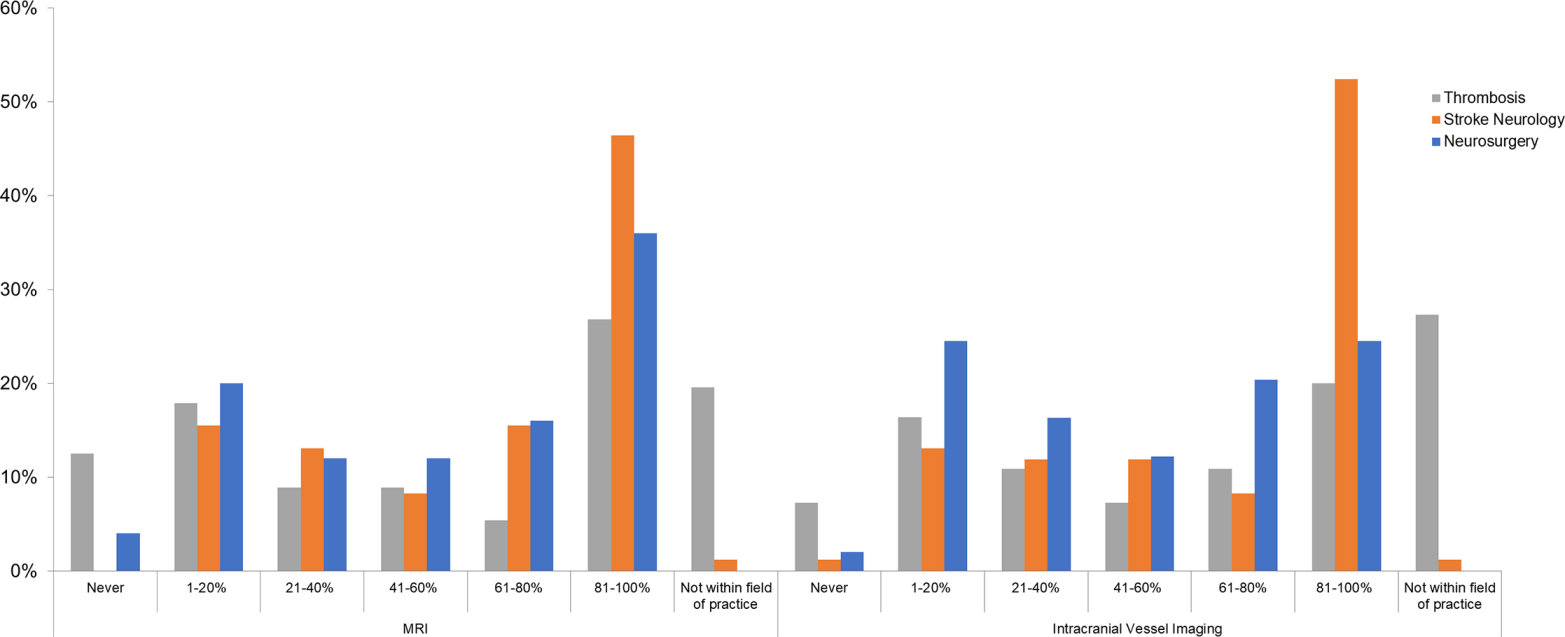
Choice of anticoagulant for re-initiation across thrombosis experts, stroke neurologists and neurosurgeons.

Overall, 39% of respondents chose apixaban as the OAC agent of choice for re-initiation post-ICH, with the 5mg BID (40%) being the most frequently chosen dosage, followed by 2.5mg BID (23%). Warfarin at INR 2–3 was chosen by 16% of respondents, while 11% preferred to continue previous regimen. Specialty-specific preferences were observed, with neurosurgery respondents preferring to continue previous regimen or starting adjusted dose warfarin, while stroke neurology and thrombosis participants opted to start patients on apixaban (p<0.001 by Cramer’s V, **Fig 2**). Participants’ years of experience or number of OAC-associated ICH cases were not correlated with differences in OAC agents chosen (p = NS by Cramer’s V); however, apixaban was more frequently selected as the OAC agent of choice following ICH among North American neurologists and thrombosis experts (76.5%) compared to those from outside of North America (37.0%, χ^2^<0.001, **S1 Fig**).

### Imaging for risk-stratification

**Fig 3:**
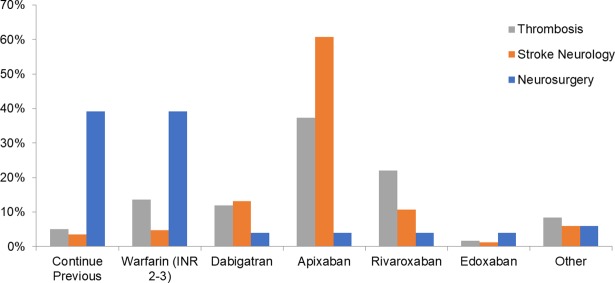
Rates of neuro-imaging utilization for risk stratification among patients with anticoagulant-associated ICH across specialties. Intracranial vessel imaging includes CT angiography, MR angiography or Digital Subtraction Angiography.

Thirty-eight percent of respondents indicated performing MRI for risk-stratification 80–100% of the time following OAC-associated ICH; this rose to 46% among stroke neurologists. Twenty percent of thrombosis experts noted use of MRI as not within their field of practice (p<0.001 across specialties for rate of MRI use, **Fig 3**). While 36% of respondents performed intracranial vessel imaging in 80–100% of OAC-associated ICH, this was 52.4% among stroke neurologists (p<0.001 across specialties for rate of intravascular vessel imaging, **Fig 3**). No impact of respondent years of experience or ICH caseload was observed for frequency of intracranial imaging. North American neurologists and thrombosis experts reported higher utilization of intracranial imaging for risk-stratification compared to those outside of North America (χ^2^<0.001, **S2 and S3 Figs**).

### Participation in research studies

In all, 71% were willing to enroll patients in a randomized controlled trial evaluating the effect of early vs. late OAC initiation following anticoagulant-associated ICH, while 56% were willing be involved in a similar capacity in a trial comparing a direct oral anticoagulant against aspirin following ICH.

## Discussion

In this study, we found a wide variation in responses regarding OAC resumption and its optimal timing following the diagnosis of ICH among patients with non-valvular AF. While there was no agreement on the timing of re-initiation, over 40% of participants preferred restarting OAC after 14 days to 3 months from the onset of ICH. Responses were heavily modified by specific clinical factors, and clinically relevant differences in decision for OAC re-initiation and neuroimaging for risk stratification were found across specialties, with neurosurgeons having earlier preferences compared to stroke neurologists or thrombosis experts across several clinical situations.

Our data suggest OAC re-initiation following ICH is a situation frequently encountered by clinicians. In a survey of 328 attendees at the 2010 AANS meeting, 48% faced dilemma on OAC re-initiation following ICH at least weekly [8]. Among AANS meeting respondents, over 30% indicated resumption of OAC after one week, whereas over 40% indicated re-starting at least one month following the incident event. Meanwhile, a survey of 329 Japanese neurosurgeons and neurologists on re-anticoagulation for non-valvular AF following ICH suggested near-equal distribution in responses for resuming anticoagulation at 5–7 days, 8–14 days or 15–28 days [9]. In comparison, our results indicate later timing of OAC re-initiation among clinicians; this may be a consequence of recent observational data suggesting excess risks of ICH recurrence associated with restarting OACs within 10 weeks of the incident ICH [10], as well as non-randomized studies suggesting all-cause mortality benefits associated OAC resumption, in which median OAC re-initiation were 31 and 34 days post-bleed [11,12].

In our survey, clinical risk factors modified the distribution of response in favor of OAC re-initiation. Most survey respondents opted to re-start OACs among most clinical scenarios, with the exception being in patients with cerebral amyloid angiopathy or in the presence of imaging previous ICH other than the index event, both known to be associated with higher rates of ICH recurrence. Meanwhile, patients with traumatic incident ICH may have lower risks, and this was reflected in earlier OAC resumption in our respondents. Notably, there was significant specialty-specific difference with respect to OAC re-initiation where neurosurgeons preferred earlier OAC re-initiation compared to stroke neurologists or thrombosis specialists. It is possible that surgical ICH cases involving OACs reflect different patient profiles that lead to lower risks of recurrence compared to non-surgical scenarios. Nonetheless, a multi-disciplinary approach to determination with respect to re-initiation of OACs, as advocated in the 2016 European Society of Cardiology guideline on atrial fibrillation, is needed [13].

Our results highlight important regional variability in two areas of decision-making following an anticoagulant-associated ICH: choice of OAC agent for resumption and neuroradiologic investigations. Apixaban was preferred by more than 75% of North American respondents in our study as the antithrombotic of choice following an anticoagulant-associated ICH, whereas those outside of the region showed preference for both rivaroxaban and apixaban. These findings complement emerging data indicating international variation from prospective global registries in prescribers’ selection of antithrombotics [14]. Furthermore, non-North American respondents reported lower utilization of neurovascular imaging for risk stratification, underscoring an important gap given the crucial role of neurovascular features in determining ICH recurrence risks and informing the safety of OAC re-introduction [6]. Evaluation of underlying factors for these practice variations are therefore key areas of additional research.

Compared to its spontaneous counterpart, warfarin-associated ICH carries larger hematoma volumes, higher risk of hematoma expansion, and portends worse clinical outcomes [15,16]. While non-vitamin K antagonist oral anticoagulants have been shown to halve the risk of ICH compared to warfarin, absolute risks with these agents remain between 0.1 and 0.26 cases per 100 person-years [17]. This residual risk, combined with lower threshold for anticoagulant initiation and broader target population for treatment, highlights the reality that ICH will remain a feared complication of OAC treatment. Importantly, history of ICH was an exclusion criterion in all trials involving direct oral anticoagulants. Recently, a Cochrane systematic review on antithrombotic therapy after ICH identified only two randomized trials involving 121 participants [18], while a similar meta-analysis of observational studies showed resumption of anticoagulation resulted in lower rates of recurrent ischemic strokes without the cost of increased ICH recurrence [19]. This highlights the urgency of ongoing prospective, randomized RESTART (ISRCTN71907627)/SoSTART (EudraCT 2016-004121-16), APACHE-AF (NCT02565693) and NASPAF-ICH (NCT02998905) trials to support clinical decision-making in this area.

Our study is subject to several limitations. First, the nature of a survey requires respondents to answer theoretical clinical scenarios that may not encompass the full granularity of real-life clinical practice. To minimize this risk, we included clinical risk factors modifiers and found consistent changes in survey response. Second, our results may be affected by non-responder bias. While our response rates are similar to those typically observed in similar international stroke surveys [20,21], selection bias cannot be ruled out. Nonetheless, our cross-sectional survey captured a representative sample of practicing neurosurgeons, stroke neurologists and thrombosis experts worldwide facing ICH in patients with non-valvular AF, and obtained diverse distribution of responses in countries, years in practice, and number of anticoagulant-associated ICH cases per year. Furthermore, participant responses were consistent with those observed in cohort studies of real-world practice [10–12], reflecting the lack of high-quality evidence in challenging clinical decisions where the balance between risk of thromboembolism and bleeding must be weighed. Finally, left atrial appendage closure was not included as an option in our survey. However, the efficacy of these devices remains uncertain outside the context of well-selected patients in randomized trials [22], and need for OAC treatment followed by dual antiplatelet therapy after device implantation during device endothelialization [23] makes the issue of anticoagulant resumption equally relevant in this population.

In summary, through a cross-sectional survey of clinicians across three specialties involved in the care of non-valvular AF patients with ICH, we demonstrated wide variation in the current practice when clinicians are faced with the dilemma of re-initiating patients on OACs following ICH. These decisions were influenced by factors related to both the patient and provider. Our observed variation likely reflects the immense gap in current evidence and this is supported by recent guidelines [13]. Data from prospective randomized trials in this population are therefore urgently needed to provide optimal care in this group of patients who share high thrombotic and recurrent hemorrhagic risks.

## Supporting information

S1 Fig Choice of anticoagulant for re-initiation between North American and non-North American respondents. (TIF)

S2 Fig Rates of magnetic resonance imaging for risk stratification following anticoagulant-associated ICH between North American and non-North American respondents. (TIF)

S3 Fig Rates of intracranial vessel imaging for risk stratification following anticoagulant-associated ICH between North American and non-North American respondents. Intracranial vessel imaging includes CT angiography, MR angiography or Digital Subtraction Angiography.
(TIF)

S1 File Survey questions disseminated to participants. (DOCX)
