# Correction: Oral anticoagulant re-initiation following intracerebral hemorrhage in non-valvular atrial fibrillation: Global survey of the practices of neurologists, neurosurgeons and thrombosis experts

**DOI:** 10.1371/journal.pone.0198031

**Published:** 2018-05-24

**Authors:** Yan Xu, Ashkan Shoamanesh, Sam Schulman, Dar Dowlatshahi, Rustam Al-Shahi Salman, Ioana Doina Moldovan, Philip Stephen Wells, Fahad AlKherayf

The fifth author is listed incorrectly in the citation. The correct citation is: Xu Y, Shoamanesh A, Schulman S, Dowlatshahi D, Al-Shahi Salman R, Moldovan ID, et al. (2018) Oral anticoagulant re-initiation following intracerebral hemorrhage in non-valvular atrial fibrillation: Global survey of the practices of neurologists, neurosurgeons and thrombosis experts. PLoS ONE 13(1): e0191137. https://doi.org/10.1371/journal.pone.0191137.

An incorrect file was used for Fig 1. Please see the complete, correct Fig 1 here.

**Fig 1:**
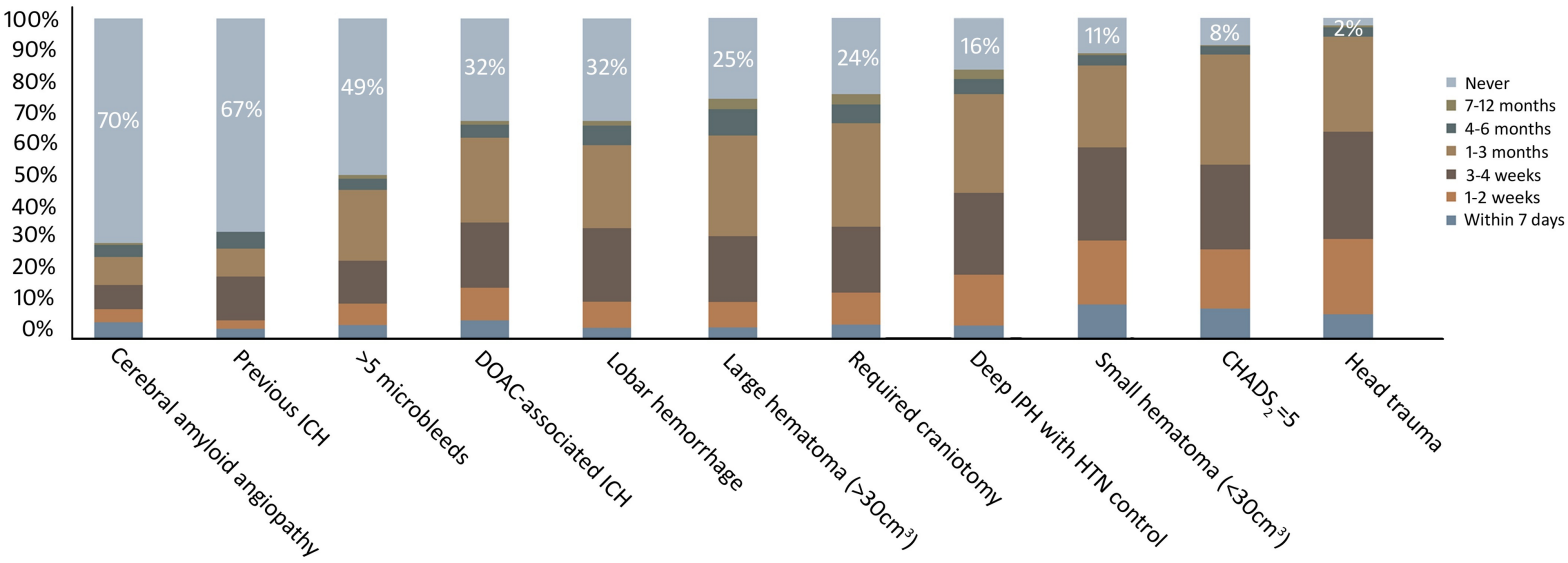
Overall response from survey participants on timing of OAC re-initiation across 11 clinical scenarios. ICH, intracerebral haemorrhage; DOAC, direct oral anticoagulants; IPH, intraparenchymal haemorrhage; HTN, hypertension; CHADS2, Congestive heart failure, hypertension, age (≥75), diabetes, stroke/TIA score.

The images for Figs 2 and 3 are incorrectly switched. The image that appears as Fig 2 should be Fig 3, and the image that appears as Fig 3 should be Fig 2. The figure captions appear in the correct order.

**Fig 2:**
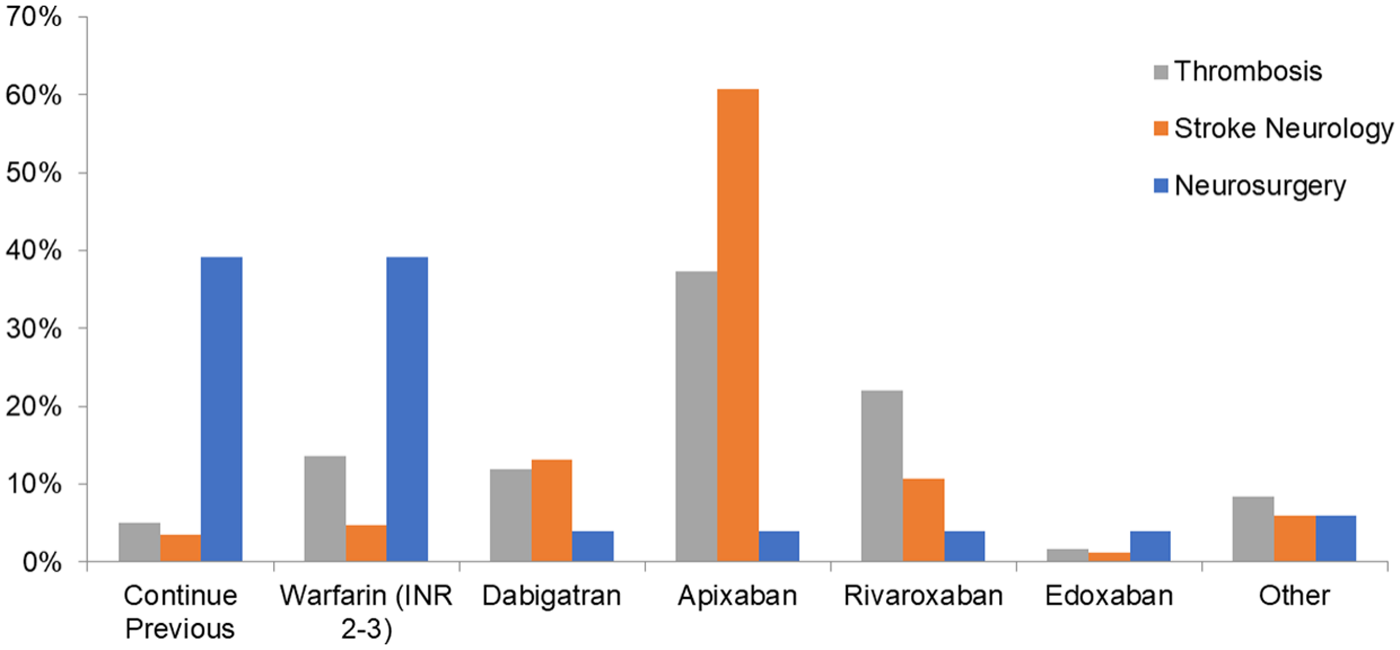
Choice of anticoagulant for re-initiation across thrombosis experts, stroke neurologists and neurosurgeons.

**Fig 3:**
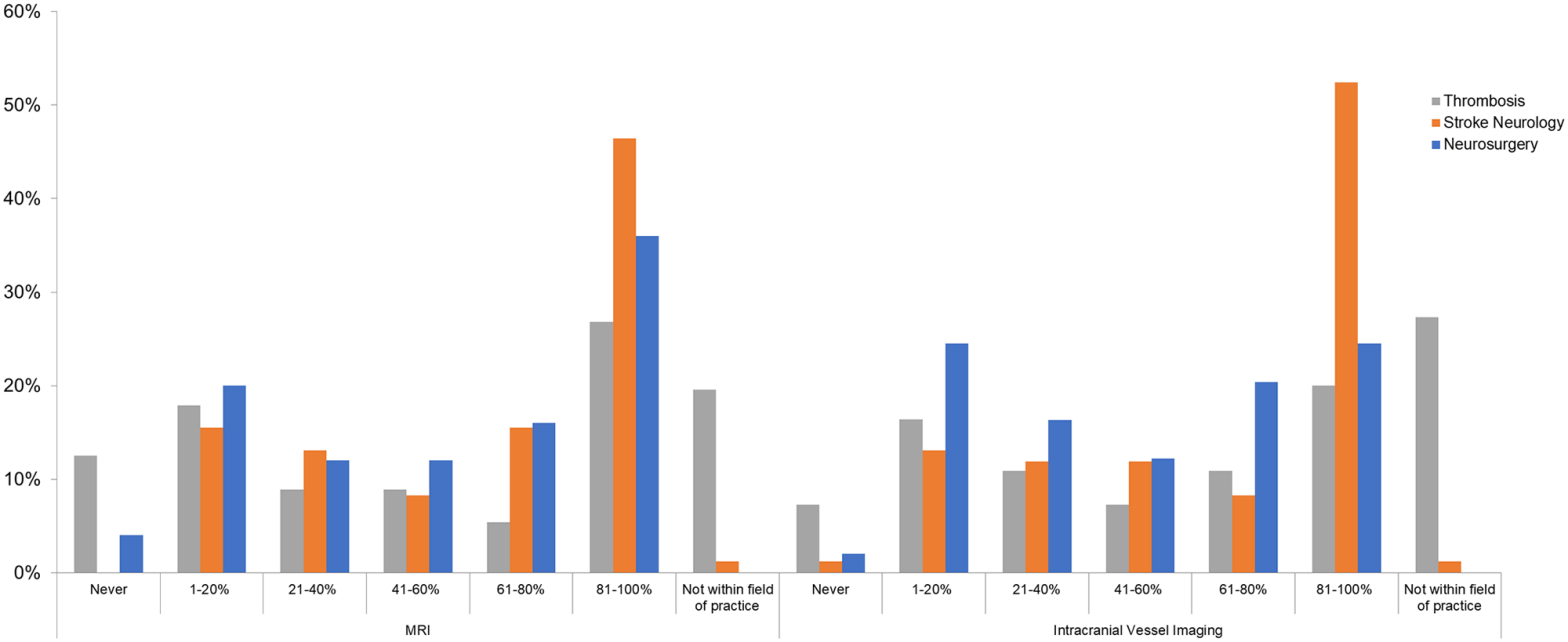
Rates of neuro-imaging utilization for risk stratification among patients with anticoagulant-associated ICH across specialties. Intracranial vessel imaging includes CT angiography, MR angiography or Digital Subtraction Angiography.
