# Supplementary figures and images for: Oral anticoagulant re-initiation following intracerebral hemorrhage in non-valvular atrial fibrillation: Global survey of the practices of neurologists, neurosurgeons and thrombosis experts
Source: PLoS One. 2018 Jan 25;13(1):e0191137. doi: 10.1371/journal.pone.0191137 (PMC5784940; doi:10.1371/journal.pone.0191137)

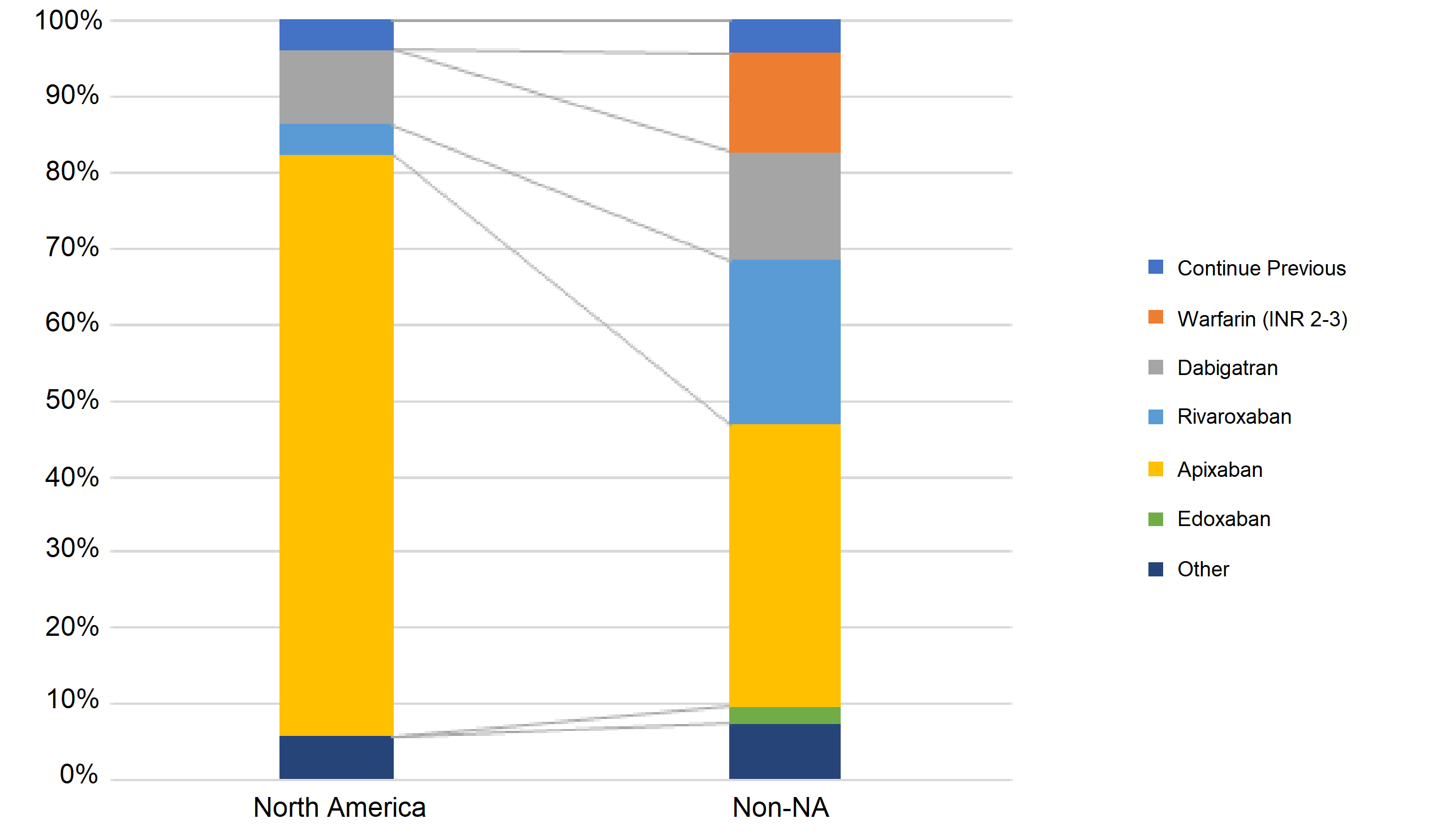

Supplement: S1 Fig — (TIF) [file pone.0191137.s001.tif]

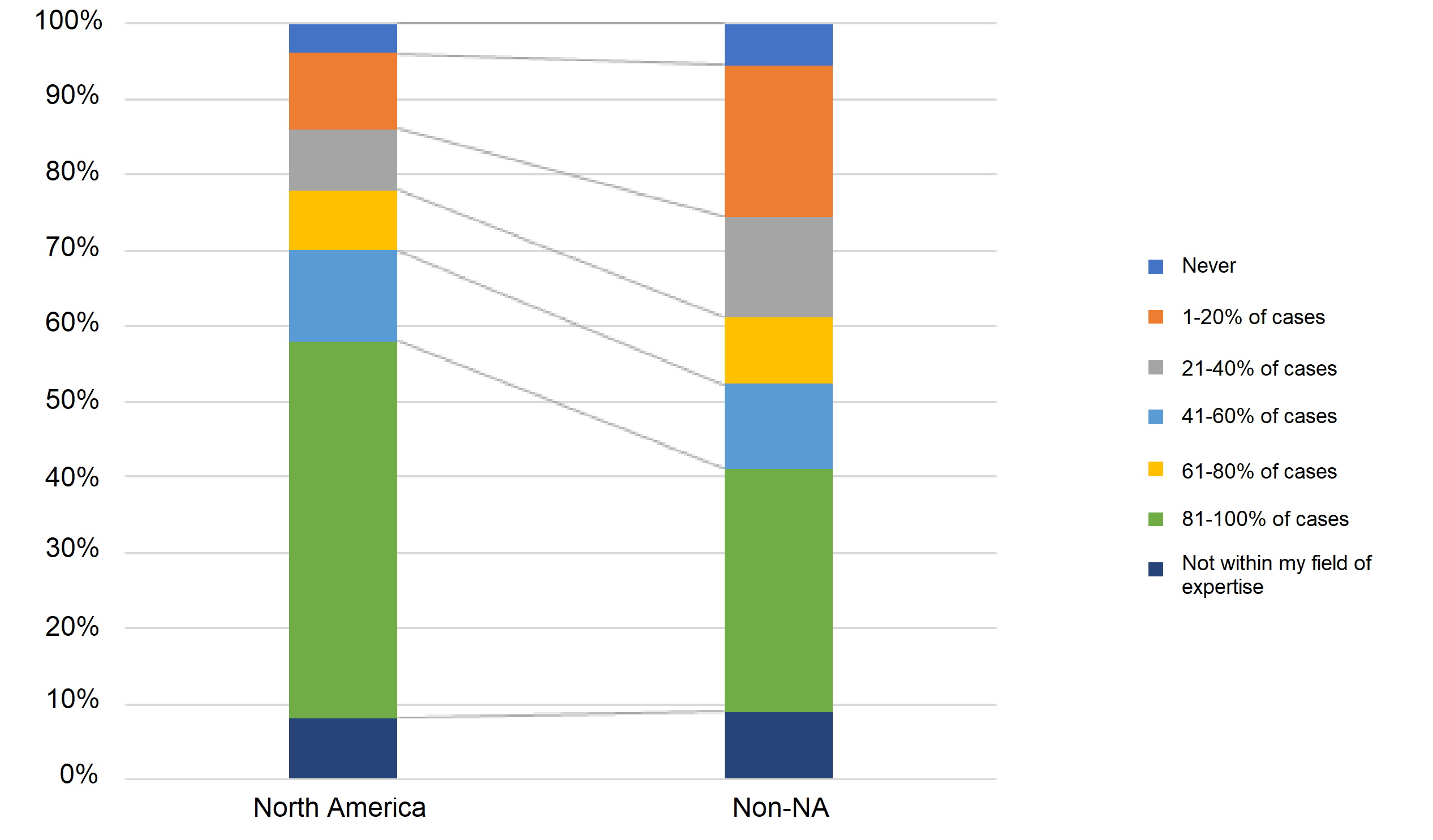

Supplement: S2 Fig — (TIF) [file pone.0191137.s002.tif]

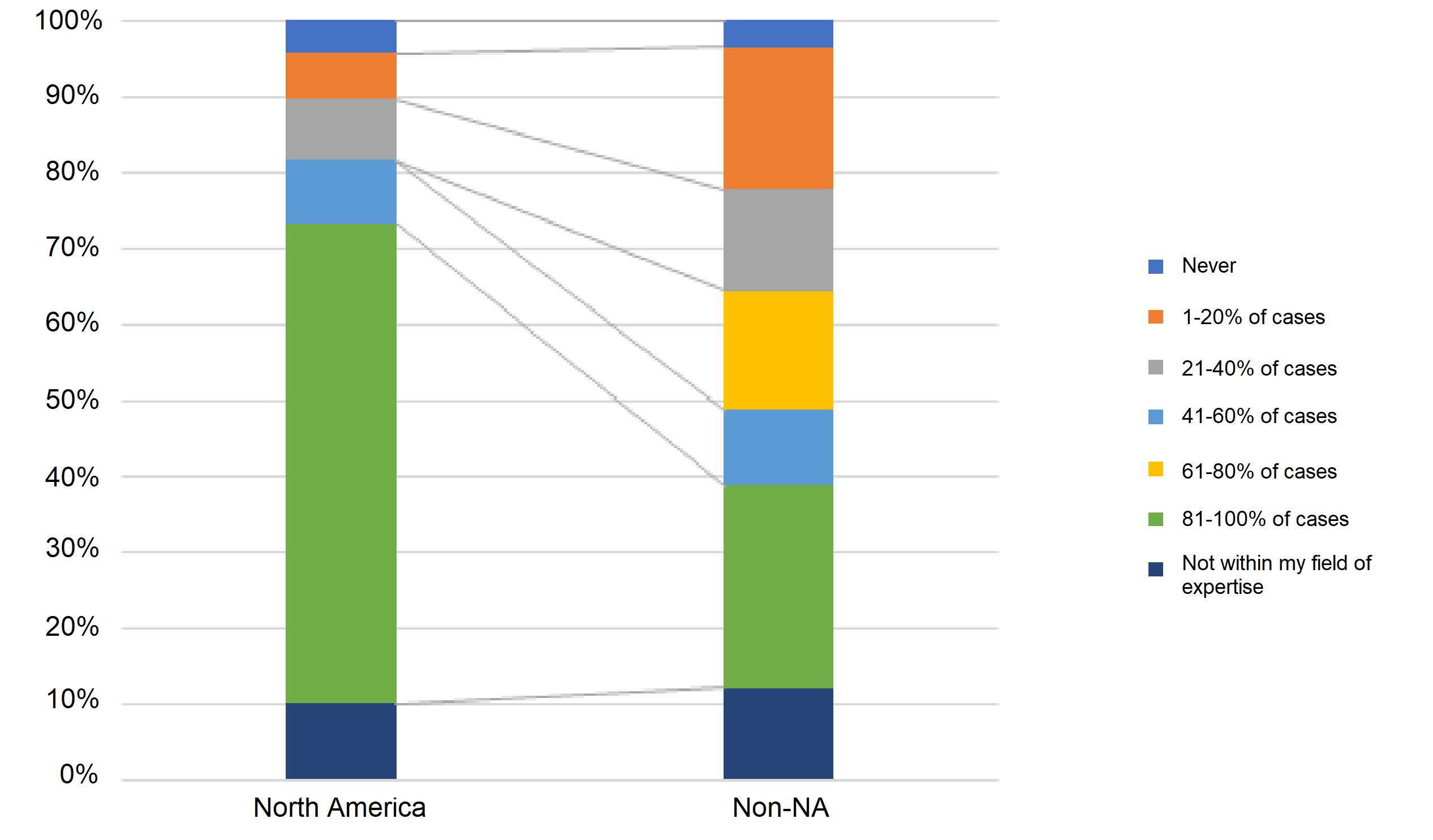

Supplement: S3 Fig — Intracranial vessel imaging includes CT angiography, MR angiography or Digital Subtraction Angiography. (TIF) [file pone.0191137.s003.tif]
