# Supplementary material for: Oral anticoagulant re-initiation following intracerebral hemorrhage in non-valvular atrial fibrillation: Global survey of the practices of neurologists, neurosurgeons and thrombosis experts
Source: PLoS One. 2018 Jan 25;13(1):e0191137. doi: 10.1371/journal.pone.0191137 (PMC5784940; doi:10.1371/journal.pone.0191137)
Supplement: S1 File — (DOCX) [file pone.0191137.s004.docx]

**Appendix**. Survey Questions

Anticoagulant Re-Initiation Post-Intraparenchymal Hemorrhage

# Q.1: What is your specialty?

|  | Neurosurgery |
| --- | --- |
|  | Neurology |
|  | Thrombosis |
|  | Other, please specify... ______________________ |

# Q. 2: How many years have you been in practice?

|  | 0-5 years |
| --- | --- |
|  | 6-10 years |
|  | 11-15 years |
|  | 16-20 years |
|  | 21-25 years |
|  | >25 years |

# Q. 3: On average, how many cases of anticoagulant-associated intraparenchymal hemorrhage in patients with non-valvular atrial fibrillation do you see every year?

|  | 0 |
| --- | --- |
|  | 1-5 |
|  | 6-10 |
|  | 11-15 |
|  | >15 |

# Q. 4: What is your country of practice?

|  | USA |
| --- | --- |
|  | Canada |
|  | Other, please specify... ______________________ |

# Q. 5: What type of practice do you have?

|  | University hospital |
| --- | --- |
|  | Hospital with university affiliation |
|  | Community hospital |
|  | Private clinic |
|  | Other, please specify... ______________________ |

# Q.6: Consider the following clinical scenario:

A 72-year old male patient with non-valvular atrial fibrillation and a CHADS2 score of 2, who is taking warfarin presents with an intraparenchymal haemorrhage (IPH). He recovers and is being discharged.

# When would you restart his oral anticoagulant from the time of IPH, given the following information?

| If this patient required a craniotomy (or surgical evacuation of the haematoma) | \|  \| Within 7 days \| \| --- \| --- \| \|  \| 1 - 2 weeks \| \|  \| 3 - 4 weeks \| \|  \| 1 - 3 months \| \|  \| 4 - 6 months \| \|  \| 7 - 12 months \| \|  \| Never \| |
| --- | --- | --- | --- | --- | --- | --- | --- | --- | --- | --- | --- | --- | --- | --- | --- |
| If this patient had a small haematoma (<30 cm3) | \|  \| Within 7 days \| \| --- \| --- \| \|  \| 1 - 2 weeks \| \|  \| 3 - 4 weeks \| \|  \| 1 - 3 months \| \|  \| 4 - 6 months \| \|  \| 7 - 12 months \| \|  \| Never \| |
| If this patient had a large haematoma (>30 cm3) | \|  \| Within 7 days \| \| --- \| --- \| \|  \| 1 - 2 weeks \| \|  \| 3 - 4 weeks \| \|  \| 1 - 3 months \| \|  \| 4 - 6 months \| \|  \| 7 - 12 months \| \|  \| Never \| |

# Q.6: Consider the following clinical scenario (continued)

A 72-year old male patient with non-valvular atrial fibrillation and a CHADS2 score of 2, who is taking warfarin presents with an intraparenchymal haemorrhage (IPH). He recovers and is being discharged.

# When would you restart his oral anticoagulant from the time of IPH, given the following information? (Continued)

| If the cause of bleeding was head trauma | \|  \| Within 7 days \| \| --- \| --- \| \|  \| 1 - 2 weeks \| \|  \| 3 - 4 weeks \| \|  \| 1 - 3 months \| \|  \| 4 - 6 months \| \|  \| 7 - 12 months \| \|  \| Never \| |
| --- | --- | --- | --- | --- | --- | --- | --- | --- | --- | --- | --- | --- | --- | --- | --- |
| If the patient had a lobar hemorrhage | \|  \| Within 7 days \| \| --- \| --- \| \|  \| 1 - 2 weeks \| \|  \| 3 - 4 weeks \| \|  \| 1 - 3 months \| \|  \| 4 - 6 months \| \|  \| 7 - 12 months \| \|  \| Never \| |
| If the patient had deep IPH with adequately controlled hypertension | \|  \| Within 7 days \| \| --- \| --- \| \|  \| 1 - 2 weeks \| \|  \| 3 - 4 weeks \| \|  \| 1 - 3 months \| \|  \| 4 - 6 months \| \|  \| 7 - 12 months \| \|  \| Never \| |
| If imaging showed >5 mixed/diffuse cerebral microbleeds | \|  \| Within 7 days \| \| --- \| --- \| \|  \| 1 - 2 weeks \| \|  \| 3 - 4 weeks \| \|  \| 1 - 3 months \| \|  \| 4 - 6 months \| \|  \| 7 - 12 months \| \|  \| Never \| |

# Q.6: Consider the following clinical scenario (continued)

A 72-year old male patient with non-valvular atrial fibrillation and a CHADS2 score of 2, who is taking warfarin presents with an intraparenchymal haemorrhage (IPH). He recovers and is being discharged.

# When would you restart his oral anticoagulant from the time of IPH, given the following information? (Continued)

| If imaging showed evidence of cerebral amyloid angiopathy (e.g., >5 strictly lobar microbleeds, convexal SAH/cortical superficial siderosis) | \|  \| Within 7 days \| \| --- \| --- \| \|  \| 1 - 2 weeks \| \|  \| 3 - 4 weeks \| \|  \| 1-3 months \| \|  \| 4-6 months \| \|  \| 7-12 months \| \|  \| Never \| |
| --- | --- | --- | --- | --- | --- | --- | --- | --- | --- | --- | --- | --- | --- | --- | --- |
| If the patient had a CHADS2 score of 5 | \|  \| Within 7 days \| \| --- \| --- \| \|  \| 1 - 2 weeks \| \|  \| 3 - 4 weeks \| \|  \| 1-3 months \| \|  \| 4-6 months \| \|  \| 7-12 months \| \|  \| Never \| |
| If the patient had a previous intracranial hemorrhage | \|  \| Within 7 days \| \| --- \| --- \| \|  \| 1 - 2 weeks \| \|  \| 3 - 4 weeks \| \|  \| 1-3 months \| \|  \| 4-6 months \| \|  \| 7-12 months \| \|  \| Never \| |
| If the patient had the hemorrhage while on a direct oral anticoagulant (dabigatran, rivaroxaban, apixaban, edoxaban)? | \|  \| Within 7 days \| \| --- \| --- \| \|  \| 1 - 2 weeks \| \|  \| 3 - 4 weeks \| \|  \| 1-3 months \| \|  \| 4-6 months \| \|  \| 7-12 months \| \|  \| Never \| |

# Q. 7 If you decide to restart oral anticoagulation, which agent would you select?

|  | Continue what they were taking previously |
| --- | --- |
|  | Adjusted dose warfarin (INR 2-3) |
|  | Dabigatran (Dosage if preferred: ______________________ |
|  | Apixaban (Dosage if preferred: ______________________ |
|  | Rivaroxaban (Dosage if preferred: ______________________ |
|  | Edoxaban (Dosage if preferred: ______________________ |
|  | Other, please specify... ______________________ |

# Question 8. How often do you perform the following?

| Clinical MRI in workup of intraparenchymal hemorrhage (IPH)? | \|  \| Never \| \| --- \| --- \| \|  \| 1-20% of cases \| \|  \| 21-40% of cases \| \|  \| 41-60% of cases \| \|  \| 61-80% of cases \| \|  \| 81-100% of cases \| \|  \| Not within my field of practice \| |
| --- | --- | --- | --- | --- | --- | --- | --- | --- | --- | --- | --- | --- | --- | --- | --- |
| Intracranial vessel imaging (CT Angiography, MR Angiography, Digital Subtraction Angiography) in patients with IPH? | \|  \| Never \| \| --- \| --- \| \|  \| 1-20% of cases \| \|  \| 21-40% of cases \| \|  \| 41-60% of cases \| \|  \| 61-80% of cases \| \|  \| 81-100% of cases \| \|  \| Not within my field of practice \| |

# Q. 10: Would you be willing to have your AF patients participate in a randomized controlled trial comparing NOAC vs. ASA for patients following an IPH?

|  | Yes |
| --- | --- |
|  | No |

# Q. 9: Would you be willing to have your AF patients participate in a randomized controlled trial comparing early vs. later re-initiation of oral anticoagulation following IPH?

|  | Yes |
| --- | --- |
|  | No |

# Question 10 Please share with us any feedback/comments

# Thank you for your time!
